# The maintenance effect of acupuncture on the side effects of breast cancer endocrine therapy: A protocol for systematic review and meta analysis

**DOI:** 10.1097/MD.0000000000020567

**Published:** 2020-06-12

**Authors:** Kejin Shi, Ying Tang, Fengyi He, Xiao Xiao, Jiayuan Zhang, Yuxia Jin, Yunxia Wang, Qi Zhang

**Affiliations:** Chengdu university of traditional Chinese medicine, Chengdu, Sichuan province, China.

**Keywords:** acupuncture, breast cancer, side effects, systematic review protocol

## Abstract

**Background:**

Breast cancer is common among women throughout the world and endocrine therapy is an established part of its treatment. But, unfortunately, this has also resulted in intolerable side effects affecting the quality of life. Acupuncture has been widely used to treat endocrine-related side effects in patients with breast cancer, but how long its effect can be maintained has not been published. The systematic review is designed to evaluate the maintenance efficacy of acupuncture for related side effects after breast cancer endocrine therapy.

**Methods and analysis:**

We will search for the following databases: PubMed, Embase, Cochrane Library, Web of Science, including China National Knowledge Infrastructure (CNKI), WanFang Data, Technology Periodical Database (VIP), and China Biology Medicine (CBM) from inception to May 2020. Two reviewers will search these databases, collect all articles, and assess the quality of studies separately, and there will be no limitations on language. The primary outcomes will be assessed using acupuncture for endocrine-related hot flashes and joint pain duration (1 month, 3 months, 6 months). Measurement tools include the Kupperman index, Brief Pain Inventory Short Form (BPI-SF), the Western Ontario and McMaster Universities Osteoarthritis Index (WOMAC), the Brief Pain Inventory-Short (BPI-SF). We will use RevMan V.5.3 for meta-analysis and employ the Grading of Recommendations Assessment, Development and Evaluation System to assess the quality of evidence.

**Results:**

This systematic review will evaluate the maintenance efficacy of acupuncture on the side effects of breast cancer endocrine therapy.

**Conclusion:**

This study will provide high-quality current evidence of how long its effect can be maintained after acupuncture for related side effects after breast cancer endocrine therapy.

**Ethics and dissemination:**

Ethical committee approval is not required for this systematic review as patient data will not be collected. This study will help to inform doctors and researchers on the duration of acupuncture treatment for endocrine-related hot flashes and joint pain. The results will be published in a peer-reviewed journal and will be disseminated in relevant conferences.

**INPLASY registration number:**

INPLASY202040024

## 1 Introduction

Breast cancer is the most common type of cancer among women worldwide, and its rate of incidence is annually increasing.^[1]^ By 2050, the incidence of breast cancer is estimated to reach 3.2 million,^[2]^ Hormone receptors positive breast cancer is the most common type of breast cancer, The National Comprehensive Cancer Network (NCCN) guidelines state that hormone receptor-positive breast cancer patients should be given endocrine therapy to reduce the recurrence and metastasis of breast cancer patients by inhibiting the secretion of estrogen in the body.^[3]^ It has been shown that endocrine therapy (ET) can reduce the recurrence rate of breast cancer by about 50%.^[4]^ Therefore, endocrine therapy plays an important role in breast cancer. However, it is also associated with numbers of side effects, including vasomotor symptoms, musculoskeletal, and vulvovaginals symptoms, the most frequent and discomfiting side effects of ET is the hot flashes and joint pain. Evidence shows that the incidence of arthralgia caused by breast cancer endocrine therapy is about 50%, and the incidence of menopausal hot flashes is as high as 50% to 60%.^[5]^ Patients with breast cancer have a high-frequency of terrible hot flashes than other healthy postmenopausal women.^[6]^ Hot flashes are characterized by a sudden feeling of warmth, and even intense heat spreads throughout the body, and ET often aggravates hot flashes, disturbing activities, sleep and eventually leading to poor quality of life.^[7]^ Aromatase inhibitor-induced arthralgia usually affects the small joints in the extremities; pain is often worse when sedentary, and relieved when physically active.^[8]^ This syndrome is significantly disadvantaged to women's quality of life, and result in many women does not complete their prescribed course of therapy.^[9,10]^ These uncomfortable affect the quality of life, treatment adherence and even impact on cancer outcomes.^[11,12]^ Although many strategies have been used to manage these symptoms, there is still no standard, thence there is a need for safe and effective interventions.^[13]^

Acupuncture is a traditional Chinese therapy that involves the insertion of single-use needles into acupoints, has been widely used to treat various conditions because it is safe and effective,^[14]^ A lot of patients with breast cancer wish acupuncture can be included in their treatment plan, even some U.S. cancer research institutions recommend acupuncture as an important means to improve the symptoms of breast cancer patients,^[15]^ In some previous researches, acupuncture has been shown to be good effect on hot flashes caused by endocrine therapy in breast cancer, including a positive effect on the severity and frequency of hot flashes,^[16]^ but little adverse effects for managing hot flashes in breast cancer survivors, the mechanisms of acupuncture may change the levels of neurotransmitters, and modulating neuroendocrine network.^[17]^ Similarly, evidence shows that acupuncture can stimulate the secretion of endorphins and P substances in the body,^[18]^ to reduce joint pain,^[19]^ including good relief of pain, joint stiffness,^[20]^ and related symptoms caused by joint pain.^[21]^ In prior studies evaluated acupuncture for hot flashes persistent effects following completion of the intervention, showing that the effects may be in 3 to 6 months, and for arthralgias about 1 to 2 months.^[16]^ However, these studies are small sample tests. Several systematic reviews of acupuncture on endocrine-related side effects were reported but did not evaluate maintenance or durability of effect, which play an important role in guiding the clinical treatment.^[22,23]^

The aim of this meta-analysis, therefore, was main to assess the maintenance efficacy of acupuncture for treatment-related hot flashes and arthralgias of hormone therapy on patients with breast cancer.

## 2 Methods

### 2.1 Study registration

The protocol for this systematic review was registered on INPLASY (Unique ID number), and is available in full on the inplasy.com (https://doi.org/10.37766/inplasy00000000). The registration number: INPLASY202040024. This protocol is structured and reported in accordance with the Preferred Reporting Items for Systematic Reviews and Meta-Analyses Protocols (PRISMA-P) statement guidelines.^[24]^ The review will be operated according to the Preferred Reporting Items for Systematic Reviews and Meta-Analyses (PRISMA) statement guidelines.^[25]^

### 2.2 Inclusion criteria for study selection

#### 2.2.1 Type of study

We will include randomized controlled trials (RCTs) of acupuncture therapy for endocrine-related hot flashes and arthralgias in patients with breast cancer. No language is limited. We will remove Non-RCTs, case series, reviews, duplicate publications, and animal studies.

#### 2.2.2 Types of participants

Patients diagnosed with breast cancer and symptoms of hot flashes and arthralgias induce by ET will be included. There will be no restrictions on age, tumor stage education, ethnicity.

#### 2.2.3 Type of intervention

The treatment group should be treated with manual acupuncture or electroacupuncture, and acupuncture points and treatment duration are not limited. The control group should adopt one of the following treatment methods: western medicine, placebo, sham acupuncture.

#### 2.2.4 Types of outcome measures

Primary outcomes

(1) Kupperman index^[26]^: This is internationally used to assess menopause symptoms. All studies should fully record the frequency and severity of hot flashes.
(2) BPI-SF^[27]^: Including 11-item scale that allows patients to rate the severity of their pain and the degree to which their pain interferes with daily functioning
(3) WOMAC^[28]^ index: The Western Ontario and McMaster Universities Osteoarthritis index is a validated measure for assessing osteoarthritis of the knees or hips and consists of 24 questions related to three subscales, including pain, stiff, and physical function.

Secondary outcomes

MenQoL^[29]^: The Menopause Quality of Life questionnaire is a tool to assess health-related quality of life in the immediate postmenopausal period

(1) HAQ^[30]^: The Health Assessment Questionnaire is a well-vali-dated tool that has been used extensively for the past 2 decades to evaluate patients with rheumatic disorders.

### 2.3 Search methods

We will search for the following databases: PubMed, Embase, Cochrane Library, Web of Science, including China National Knowledge Infrastructure (CNKI), WanFang Data, Technology Periodical Database (VIP), and China Biology Medicine (CBM) from inception to November 2019. The WHO International Clinical Trials Registration Platform and Chinese Clinical Trial Registry will also be searched to identify any ongoing experiment. We also manually retrieved and reviewed the reference lists of identified relevant RCTs, reviews and overview comments, and contacted the trial author for the ongoing RCTs clinical data. The key search terms used are breast neoplasm∗, breast cancer∗, breast tumor∗, acupuncture, randomized controlled trial. The search strategy for the PubMed database is shown in Table 1; this strategy will be modified according to other databases (Table 1 Search strategy used in PubMed).

**Table 1:**
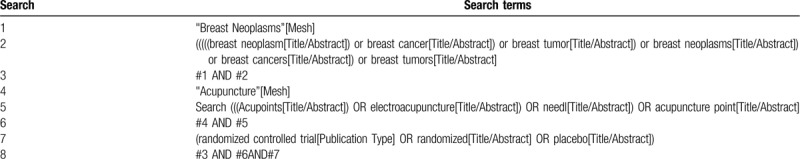
Search strategy used in PubMed.

### 2.4 Data collection and analysis

#### 2.4.1 Selection of studies

**Figure 1:**
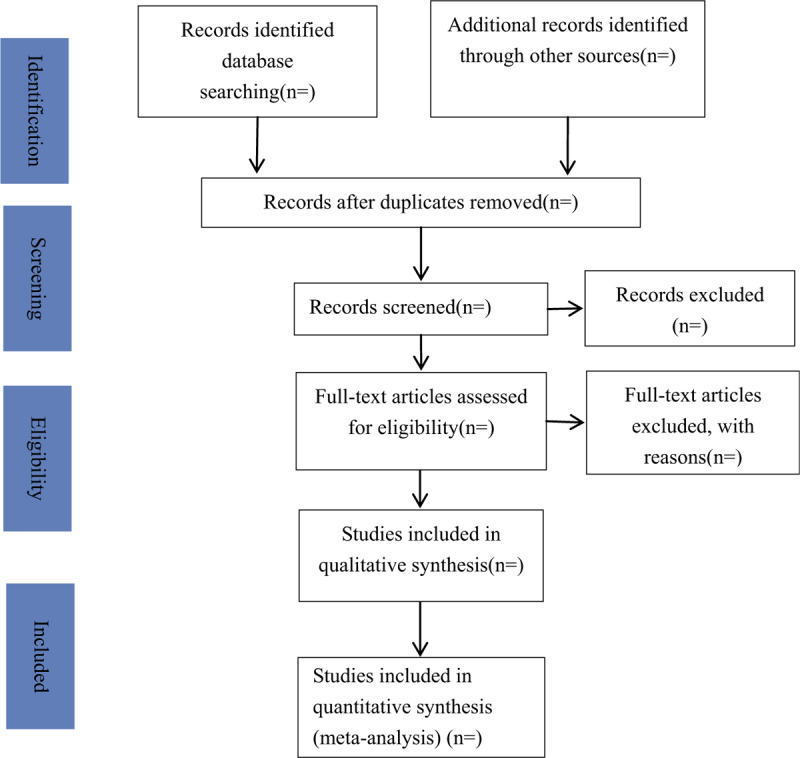
Flow chart of study selection process.

Two reviewers independently collect articles and include or exclude according to the above criteria. If there are disagreements between 2 reviewers, a third reviewer will make the final decision. If the agreement is still not reached, we will consult the expert or author in this area. The entire selection process is shown in the PRISMA flow diagram (Fig. 1).

#### 2.4.2 Data extraction and management

Two reviewers will independently extract information from the included trials, and enter the data into a predefined data extraction table. The extracted data information will include the author, publication year, title, patient age, type of intervention, the sample size of each group, intervention time, allocation concealment, randomization, blinding, the main outcome, duration of follow-up, and main points. If there is a controversy, a third reviewer will accord the best way to make the final judgment. If the data are insufficient, it can be obtained by contacting the original author for further information.

#### 2.4.3 Assessment of risk of bias

The assessments include 7 domains:

1. random sequence;
2. generation;
3. allocation concealment;
4. blinding;
5. incomplete outcome data;
6. selective reporting;
7. other biases.

Any disagreement will also be resolved by discussion.

#### 2.4.4 Measures of treatment effect

For continuous outcomes data, the mean difference, or standard mean difference will be used to measure the treatment effect with 95% confidence intervals (CIs). For dichotomous data, risk ratios with 95% CIs will be used.

#### 2.4.5 Dealing with missing data

We will try to contact the authors by email or phone to obtain any missing data if possible. If this method is unsuccessful, we will exclude these studies.

#### 2.4.6 Unit of analysis

The analysis unit will be the individual participant.

#### 2.4.7 Assessment of heterogeneity

According to the Cochrane Handbook for Systematic Reviews of Interventions, heterogeneity can be assessed by the *χ*^2^ test or *I*^2^ value, a random-effects model will be used to estimate the overall treatment effect if the *P*-value is <.10 and the *I*^2^ value is >50%, otherwise, a fixed-effects model will be used. Moreover, if there is significant heterogeneity between the studies, meta-regression or subgroup analysis will be used to explore the causes of heterogeneity among the results of studies.

#### 2.4.8 Assessment of reporting bias

The funnel plot and Egger test will be used to evaluate the publication bias if more than 10 studies are included.

#### 2.4.9 Data synthesis

Review Manager V.5.3 software will be used for the meta-analysis. Risk ratios will be used for describing the dichotomous effect size, mean difference, or standard mean difference for continuous data. The fixed-effects model will be used for data synthesis. If *I*^2^ is <50%, otherwise, a random-effects model will be used. When the meta-analysis is not appropriate, we will conduct a narrative describing the results.

#### 2.4.10 Subgroup analysis

Subgroup analyses will be performed according to the heterogeneity between the trials. The analysis will include the duration of follow-up (1 month, 3 months, 6 months etc).

#### 2.4.11 Sensitivity analysis

To test the robustness of treatment effects, the sensitivity analysis will be tested by the studies sample size, heterogeneity quality, and selected model (random-effects vs fixed-effects model).

#### 2.4.12 Grading of evidence quality

Two authors will independently use the Grading of Recommendations Assessment Development and Evaluation to assess the quality of the evidence. The quality of the results including: high, moderate, low, and very low.

## 3 Discussion

Acupuncture therapy is an effective alternative intervention for ET relates side-effects. However, how long will the effect last? There are no systematic reviews on this topic have been reported. This systematic review will provide the latest evidence on the duration of side effects associated with acupuncture for breast cancer endocrine therapy. Though this study might have some potential limitations, we believe the resulting information may provide important evidence that will benefit patients, practitioners, and clinical doctors.

## Author contributions

**Conceptualization:** Kejin Shi.

**Data curation:** Jiayuan Zhang, Fengyi He.

**Formal analysis:** Xiao Xiao, Jiayuan Zhang, Yuxia Jin.

**Investigation:** Kejin Shi.

**Methodology:** Kejin Shi.

**Project administration:** Kejin Shi., Qi Zhang.

**Software:** Yunxia Wang.

**Supervision:** Qi Zhang.

**Writing – original draft:** Kejin Shi, Ying Tang

**Writing – review & editing:** Kejin Shi.
